# Serotonin 5-HT_2A_ receptor activity mediates adipocyte differentiation through control of adipogenic gene expression

**DOI:** 10.1038/s41598-021-98970-1

**Published:** 2021-10-05

**Authors:** Bangning Yu, Diana M. Battaglia, Timothy P. Foster, Charles D. Nichols

**Affiliations:** 1 Department of Pharmacology and Experimental Therapeutics, Louisiana State University Health Science Center, 1901 Perdido Street, New Orleans, LA 70112 USA; 2 Department of Microbiology, Immunology, and Parasitology, Louisiana State University Health Science Center, 1901 Perdido Street, New Orleans, LA 70112 USA

**Keywords:** Cell signalling, Metabolism

## Abstract

Serotonin 5-HT_2_ receptors are expressed in many tissues and play important roles in biological processes. Although the 5-HT_2A_ receptor is primarily known for its role in central nervous system, it is also expressed in peripheral tissues. We have found that 5-HT_2A_ receptor antagonists inhibit human subcutaneous primary adipocyte differentiation. We also show that siRNA knockdown of the 5-HT_2A_ receptor blocks differentiation. Using gene expression analysis in combination with receptor antagonists we found that activity of 5-HT_2A_ receptors is necessary very early in the differentiation process to mediate expression of adipogenic genes, including *peroxisome proliferator-activated receptor gamma (ppar-γ), adipocyte protein 2 (aP2)*, *adiponectin*, and *serine/threonine-protein kinase 1 (sgk1)*. We show here for the first time that 5-HT_2A_ receptor activity is necessary for differentiation of human primary subcutaneous preadipocytes to adipocytes, and that 5-HT_2A_ receptor activity mediates key genes related to adipogenesis during this process. Importantly, this work contributes to a greater understanding of the adipocyte differentiation process, as well as to the role of 5-HT_2A_ receptors in peripheral tissues, and may be relevant to the development of novel therapeutic strategies targeting this receptor for the treatment of obesity related diseases.

## Introduction

Serotonin (5-hydroxytryptamine, 5-HT) is an important small molecule neurotransmitter that mediates a wide variety of biological functions. Its effects are mediated by a large superfamily, which includes seven different receptor family members (5HT_1-7_) comprised of fourteen distinct subtypes. Each of these are 7-transmembrane G-protein coupled receptors, with the exception of the 5-HT_3_ receptor, which is a ligand-gated ion channel^1^. In the central nervous system, serotonin modulates a variety of behaviors including cognition, mood, aggression, mating, and feeding^1^. Serotonin also plays very important roles in many peripheral tissues that include heart, smooth muscle, kidney, gut, fibroblasts, liver, and lymphocytes.

Numerous studies have shown that genetic mutations in serotonin receptor genes are linked to obesity and cardiovascular disease^2,3^. Of all the serotonin receptors, the 5-HT_2A_ receptor has been the most linked to behaviors and CNS function, and is the most widely expressed serotonin receptor throughout the body^4–12^. Extensive work has been performed to establish the role of 5-HT_2A_ receptors within the CNS, where there they have been shown to participate in processes such as cognition and working memory. Significantly, 5-HT_2A_ receptor function has been implicated in affective disorders such as schizophrenia, and mediates the primary effects of psychedelic drugs^13^. Serotonin 5-HT_2A_ receptors also are expressed in many peripheral tissues^14–16^. Within the vasculature, 5-HT_2A_ receptors are known to modulate vasoconstriction and inflammation^14,17,18^. Genetic mutations in this receptor have been reported to be linked to cardiovascular diseases such as hypertension in both Caucasians and Asians^7,19^.

5-HT_2A_ receptor expression and function in many other peripheral tissues remains unclear. We have previously found activation of peripheral and vascular 5-HT_2A_ receptor produces potent anti-inflammatory effects, including the prevention vascular inflammation and of asthma^15,16,20–22^. Some research has implicated 5-HT_2A_ receptor activity in metabolic processes, for example, activation of 5HT_2_ receptors with the agonist DOI (1-(2,5-dimethoxy-4-iodophenyl)-2-aminopropane), has been reported to increase plasma glucagon levels in rats^23^. This hyperglucagonemia induced by DOI is dose-dependently prevented by the 5-HT_2_ receptor antagonist ketanserin. Further, blockade of 5-HT_2_ receptors with ketanserin has been shown to reduce plasma glucose levels and weight gain in mice fed a high fat diet^24^. A different antagonist at the 5-HT_2A_ receptor, sapogrelate, has been reported to elevate adiponectin expression and to have insulin-sensitizing effects^25–27^. Taken together, these studies support the notion that 5-HT_2_ receptors likely play an important, but as yet undefined, role in obesity-related diseases, including diabetes. Elucidation of the role of 5-HT_2A_ receptors is therefore likely to provide not only crucial information about these processes, but also potentially to lead to new therapeutic avenues.

In the study of the pathological processes underlying obesity and related diseases, a common and accepted model system is the in vitro study of adipocyte differentiation, a highly dynamic and regulated process, where fibroblast-like subcutaneous preadipocytes alter cellular fate, change morphology, and accumulate lipids and fatty acids^28–31^. The adipogenic transcription factor CCAAT enhancer binding protein beta (C/EBP-β) and the peroxisome proliferator activated receptor gamma (PPAR-γ), play key roles in the complex transcriptional cascade that occurs during the differentiation process^32^. With the activation of C/EBP-β and PPAR-γ, downstream factors, including adipocyte lipid binding protein (aP2), and the adipokine adiponectin are induced, leading to triglyceride elevation, lipid droplet accumulation, and mature adipocyte formation. Blockade of *c/ebp-β* and *ppar-γ* expression results in the inhibition of adipocyte differentiation^33–35^.

Although other groups have investigated the role of 5-HT_2_ receptors in adipocyte differentiation, none have used human tissues, and the others have mostly utilized mouse 3T3-L1 fibroblast cells. Although often used as a proxy for human adipocyte generation, largely because they can be manipulated to develop into an adipocyte-like phenotype, they are not equivalent to authentic human preadipocytes and utilize different mechanisms for differentiation. For example, Kinoshita and colleagues^36^ found that during the differentiation process, 5-HT_2A_ receptor mRNA transcript levels are *downregulated*, and activity at 5-HT_2C_ receptors primarily mediates serotonin’s role in the differentiation of 3T3-L1 fibroblasts to an adipocyte-like phenotype. We have found no evidence for 5-HT_2C_ receptor expression in human preadipocytes by qRT-PCR, and there is no report in the literature of human preadipocytes expressing 5-HT_2C_ receptors. In another study using 3T3-L1 fibroblasts and db/db mice, 5-HT_2A_ receptor activation *decreased* expression of adipogenic markers like adiponectin^26^. Clearly, there are important mechanistic differences between mouse and human, and the study of human tissue is necessary to fully understand the role of serotonin in adipocyte differentiation in humans. Following up on our earlier work^37^, we present here conclusive evidence demonstrating that 5-HT_2A_ receptor activity is necessary for differentiation in a more relevant adipose model system: primary human subcutaneous preadipocyte to adipocyte differentiation.

## Results

### 5HT_2_ agonist/antagonist effects on day 2 of human adipocyte differentiation

**Figure 1:**
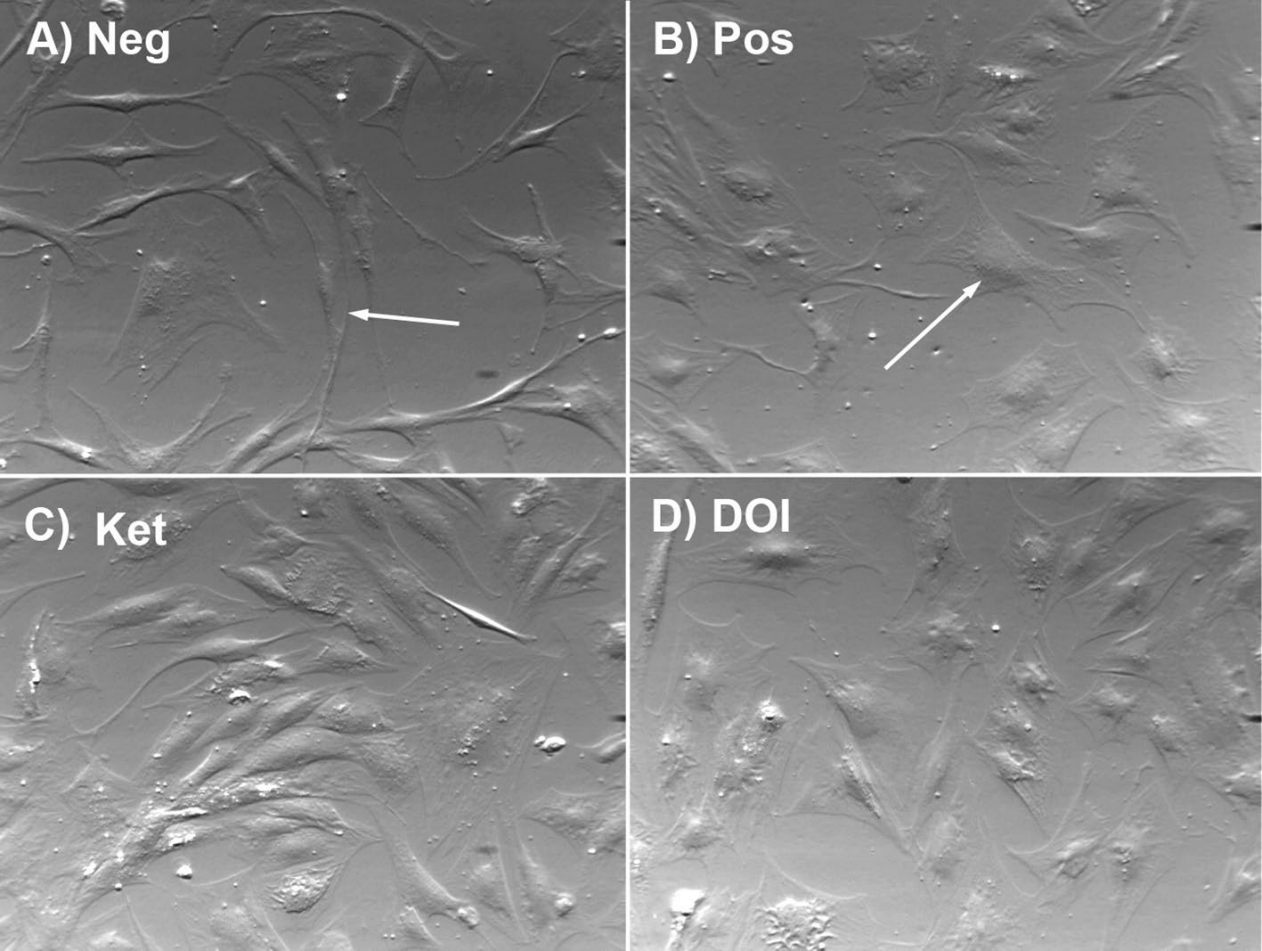
Effect of 5-HT_2_ receptor agonist/antagonist on day 2 of adipocyte differentiation. (**A**) Representative field of negative control cells maintained in basal medium. The arrow indicates a pre-adipocyte cell that has a long narrow phenotype. (**B**) Positive control cells that have been induced for 2 days have begun to change shape to a round phenotype (arrow). (**C**) Induced cells that were pre-treated with the 5-HT_2_ antagonist ketanserin (Ket) are mostly still elongated. (**D**) Induced cells pre-treated with the 5-HT_2_ agonist 2,5-dimethoxy-4-iodoamphetamine (DOI) are round in appearance similar to the positive control cells, indicating no effect on differentiation at day 2.

In order to determine the effects of 5-HT_2_ receptor activity in the early stages of adipocyte differentiation, 5-HT_2_ receptor agonist or antagonist was added to the basal medium 24 h prior to the induction medium, and added fresh with each medium change at the same concentrations. As shown in Fig. 1A on day 2, the negative control cells that were in basal medium for the entire procedure did not demonstrate any morphological changes indicative of adipocyte differentiation, whereas positive control cells in the induction media had begun to show morphological changes. As shown in Fig. 1, negative cells were cells with a long narrow phenotype, indicating that they were pre-adipocyte and had not undergone differentiation; whereas positive cells were rounded up and had a changed morphological appearance similar to differentiated adipocytes. At this time point, induced cells treated with the 5-HT_2A_ receptor antagonist ketanserin (1.0 µM) are mostly still elongated and not rounded up (Fig. 1C), indicating inhibition of differentiation. Induced cells pretreated with the 5-HT_2_ receptor agonist (*R*)-DOI (1.0 µM) were mostly rounded in appearance similar to the positive control cells (Fig. 1D).

### 5-HT_2A_ antagonist blocks terminal human adipocyte differentiation

**Figure 2:**
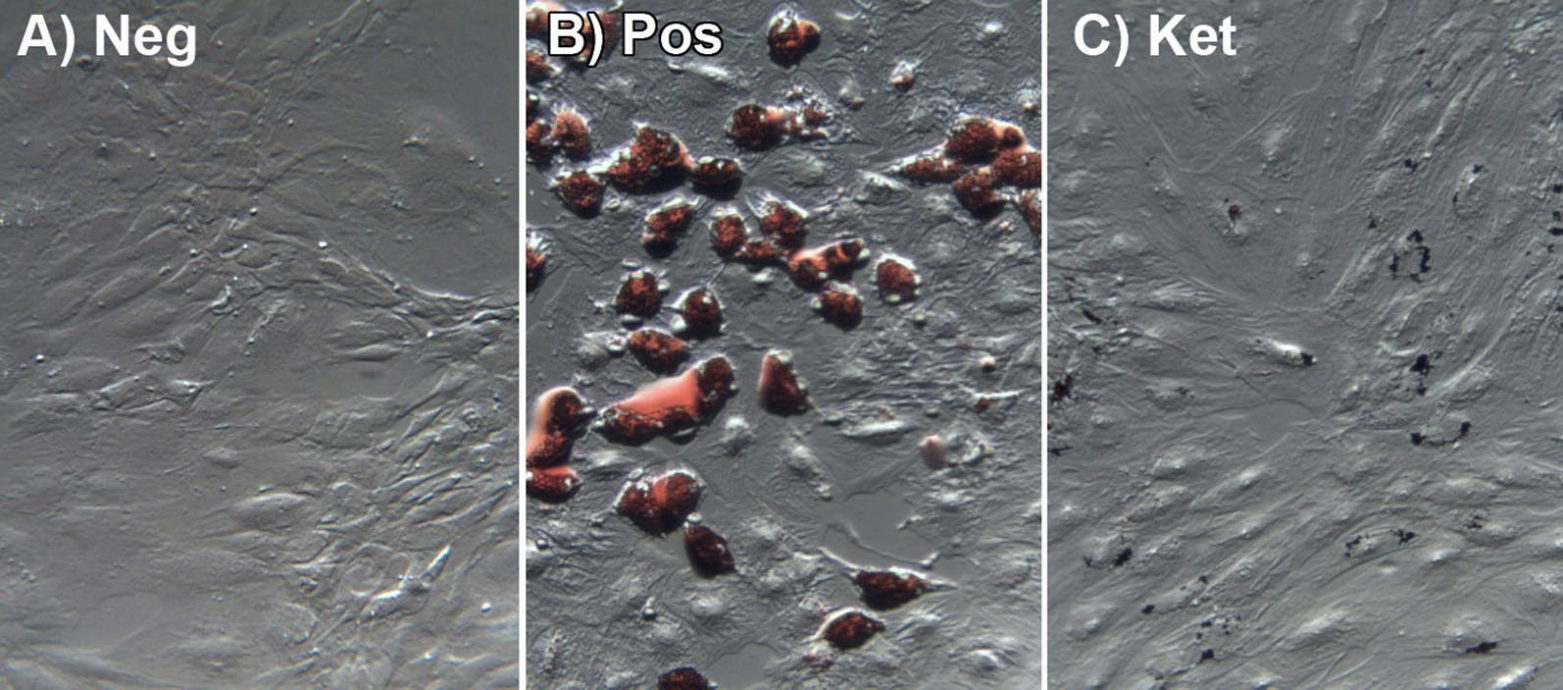
Ketanserin blocks terminal human adipocyte differentiation (Day 14). Human pre-adipocytes were differentiated for 14 days and then stained with oil red O to assess lipid accumulation as a marker for adipocyte differentiation. (**A**) Negative control cells that were maintained in basal medium for 14 days did not stain, indicating no differentiation. (**B**) Induced positive control cells stained with oil red O, indicating differentiation into mature adipocytes with lipid accumulation. (**C**) Induced positive control cells that were treated with ketanserin (1.0 µM) for 14 days showed no staining, indicating blockade of adipocyte differentiation.

To examine the effect of 5-HT_2_ receptor activity on terminal adipocyte differentiation, primary human subcutaneous pre-adipocytes were allowed to differentiate for the full 14 days and examined with oil red O staining to assess lipid accumulation. As shown in Fig. 2A, the negative undifferentiated control cells that were maintained in basal medium for 14 days retained an overall elongated morphology and demonstrated no oil red O staining, indicating the absence of lipid accumulation. Positive control cells that were treated with the differentiation media assumed a more rounded shape and accumulated lipids, as observed by staining with oil red O, indicative of successful differentiation into adipocytes (Fig. 2B). Cells treated with the differentiation protocol, plus the addition of ketanserin (1.0 µM) throughout the experiment, were similar in appearance to the undifferentiated controls, with an overall elongated shape, and no visible lipid accumulation (Fig. 2C). Induced cells treated with (*R*)-DOI throughout the process were identical in morphology to the positive control, and stained with oil red O equal to positive control levels (not shown), indicating that 5-HT_2A_ receptor activation above levels induced by serotonin normally present in the media did not alter the differentiation process. Time course experiments demonstrated that addition of ketanserin was necessary at Day 0 to inhibit differentiation completely (data not shown).

### Ketanserin dose-dependently blocks adipocyte differentiation

**Figure 3:**
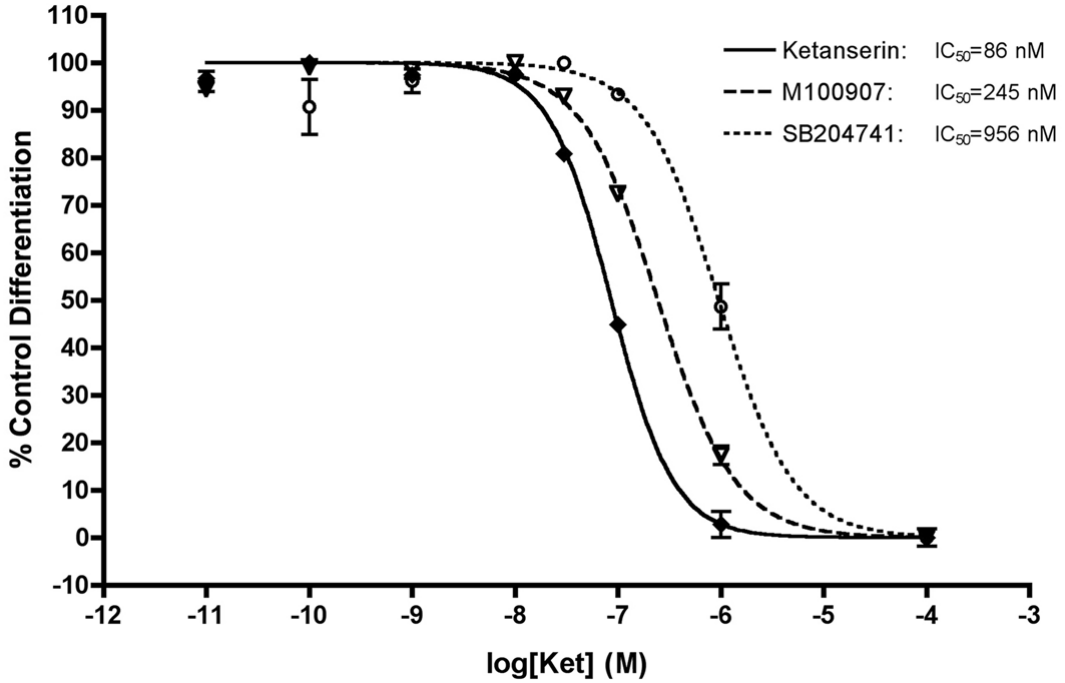
Dose–response effect of antagonists to block differentiation. The IC_50_ value of ketanserin to block human adipocyte differentiation was 86 nM. The IC_50_ values of M100907 (5-HT_2A_ selective) and SB204741 (5-HT_2B_ selective) were 245 nM and 956 nM, respectively. Oil red O staining was performed for each condition, and values compared to positive control cells for 100%, and negative control cells for 0% and presented as % control differentiation (n = 3 biological replicates for each data point ± SEM).

To examine the potency of ketanserin’s effects on adipocyte differentiation, a dose–response experiment was performed. Increasing concentrations of ketanserin were added to the cells 24 h prior to, and were maintained throughout the differentiation protocol, with terminal differentiation quantified by oil red O staining. The IC_50_ value of ketanserin to inhibit human adipocyte differentiation and lipid accumulation was determined to be 86 nM (Fig. 3). Although ketanserin is not selective for 5-HT_2A_ receptors in rodent tissues, in humans it is considered a selective 5-HT_2A_ receptor antagonist with more than 100× fold selectivity for 5-HT_2A_ over 5-HT_2C_ and 5-HT_2B_. Further, we did not detect 5-HT_2C_ receptor mRNA expression in these cells, as discussed below.

### Blockade of 5-HT_2A_ receptor activity inhibits adipocyte differentiation

To validate that the blocking effects of ketanserin are mediated by the 5-HT_2A_ receptor, dose–response experiments utilizing receptor selective antagonists for 5-HT_2A_, 5-HT_2B_, and 5-HT_2C_ receptors were performed. Antagonists used were: M100907 (5-HT_2A_), SB204741 (5-HT_2B_), and RS102221 (5-HT_2C_). Inhibition of adipocyte differentiation was observed with M100907 and SB204741 (Fig. 3). As anticipated, based upon negative QPCR expression data, the 5-HT_2C_ receptor antagonist RS102221 had no effect (data not shown). The high IC_50_ for SB204741 (956 nM) suggests that this may be an off target effect at 5-HT_2A_ receptors, but we can not rule out a contribution of 5-HT_2B_ receptors to the differentiation process (Fig. 3). These results together, as discussed later, demonstrate no involvement of 5-HT_2C_ receptors, and that activity at 5-HT_2A_ receptors likely mediates the differentiation process.

### Knockdown of 5-HT_2A_ mRNA blocks adipocyte differentiation

**Figure 4:**
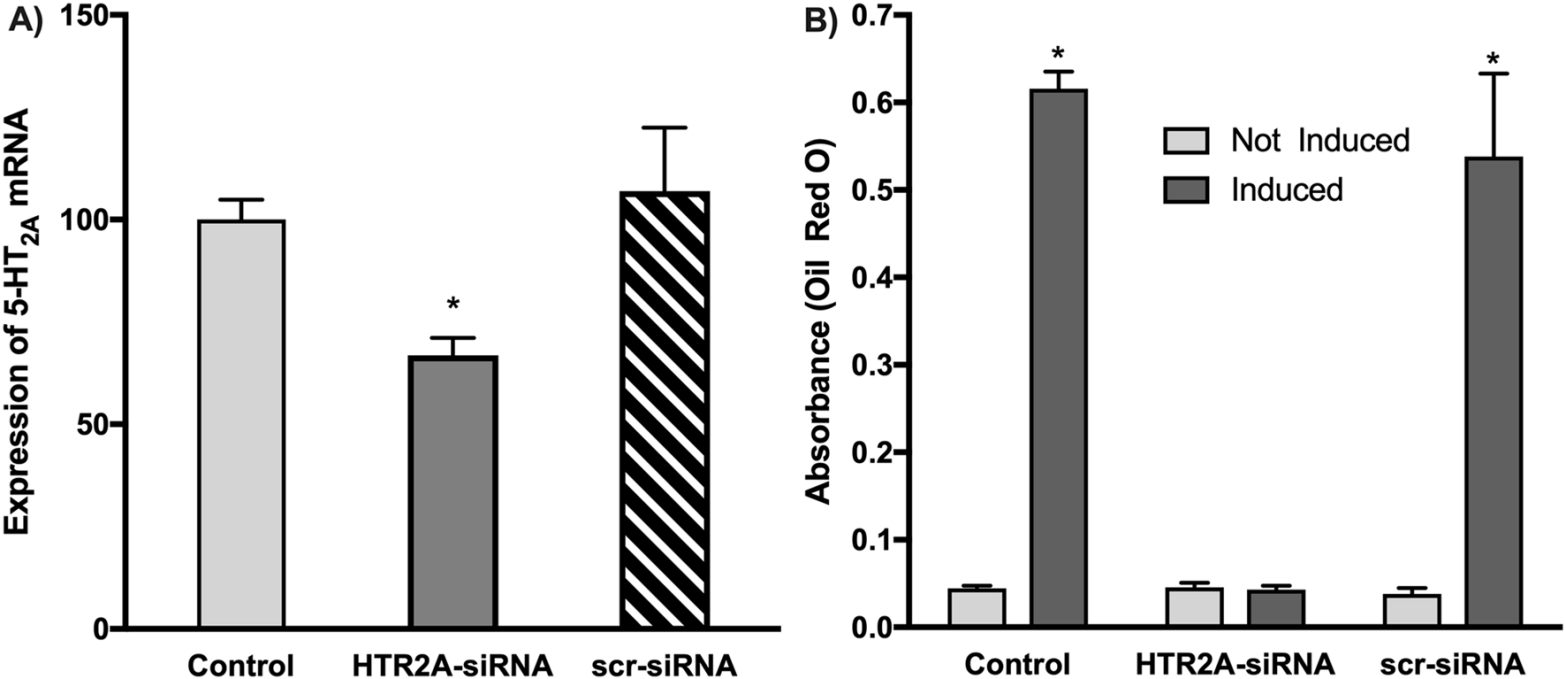
Knockdown of 5-HT_2A_ receptor mRNA blocks differentiation. (**A**) QPCR was performed on transfected cells at day 2 to determine that knockdown with the 5-HT_2A_ receptor siRNA (HTR2A-siRNA) was 30% below normal expression levels, and below levels after transfection with the scrambled siRNA (scr-siRNA). (*p > 0.05; ANOVA with Tukey post hoc test; n = 3 biological replicates per treatment, with 3 technical replicates per biological replicate per gene expression data point; error =  ± SEM). (**B**) Cells were mock-transfected (no nucleotide; control), transected with siRNA against 5-HT_2A_ receptor mRNA (HTR2A-siRNA), or transfected with the same amount of scrambled siRNA (scr-siRNA). Half of the cells were induced, while the remaining half were not induced. At day 14, the extent of differentiation was determined by oil red O staining. Only siRNA directed against 5-HT_2A_ receptor mRNA was able to block differentiation.

To definitively demonstrate involvement of 5-HT_2A_ receptor activity in the differentiation process, we used siRNA to knockdown *HTR2A* receptor mRNA expression and measured differentiation levels using oil red O staining. Here, cells were transfected with either siRNA to the 5-HT_2A_ receptor mRNA or scrambled control siRNA for 48 h prior to initiating the induction process. As shown in Fig. 4B, knockdown of the 5-HT_2A_ receptor mRNA completely blocked differentiation as quantified by oil red O staining at day 14. Neither mock-transfection of controls, nor the transfection of scrambled siRNA had any effect on levels of differentiation compered to control cells (Fig. 4B). To verify knockdown efficiency, QPCR was performed to determine that at day 2 (48 h post-transfection; day of induction) 5-HT_2A_ mRNA levels were significantly reduced by 30% compared to controls (Fig. 4A).

### Expression levels of adipocyte related genes in human primary subcutaneous preadipocytes

**Figure 5:**
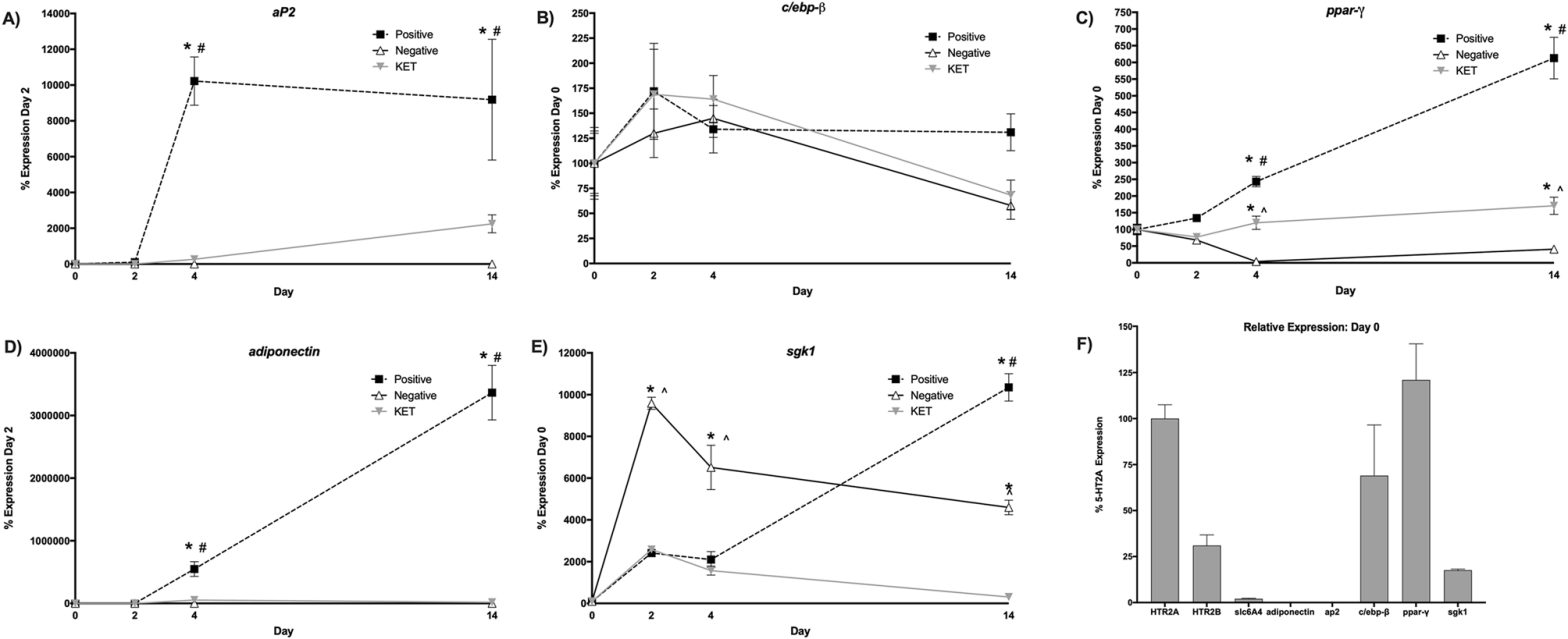
Expression of adipogenic genes are dynamic throughout the differentiation process, and are influenced by 5-HT_2A_ receptor blockade. “Positive” represents expression from controls that were induced to differentiate. “Negative” represents controls that were not induced, and were maintained in basal medium for 14 days. “KET” represents induced cells that were treated with 300 nM of Ketanserin at each step of the adipocyte differentiation process. (*p < 0.01 vs. negative control; #*p* < 0.01 vs. ketanserin treatment group; n = 3 biological replicates per treatment, with 3 technical replicates per biological replicate per gene expression data point; analysis of variance (ANOVA) with Tukey’s post hoc test). Ketanserin significantly blocks differentiation-induced expression of *ap2* (**A**), *ppar-γ* (**C**), *adiponectin* (**D**), and *sgk-1* (**E**), but does not significantly affect *c/ebp*-*β* expression (**B**). (**F**) *aP2*, *c/ebp*-*β*, *ppar-γ*, *adiponectin, HTR2A*, *HTR2B*, *SLC6A4* (serotonin transporter; SERT)*,* and *sgk-1* expression was determined in human primary subcutaneous preadipocytes by QPCR. Relative expression of these genes was analyzed using the *HTR2A* expression level as 100%. All expression experiments were performed in human primary subcutaneous preadipocyte cells at passage 5.

Q-RT-PCR experiments were performed on RNA isolated from undifferentiated human primary subcutaneous preadipocytes to determine the presence of and initial levels of each of the genes we planned to examine in the differentiation process. In undifferentiated cells, we found that 5-HT_2A_ receptor mRNA was expressed at a higher level than 5-HT_2B_ receptor mRNA. Interestingly, mRNA for the serotonin transporter gene was detected, although it was quite low compared to 5-HT_2A_ receptor mRNA expression (Fig. 5). The presence of *c/ebp-β*, *ppar-γ,* and *sgk1*, was also detected at Day 0 (Fig. 5). Although *aP2* or *adiponectin* mRNA was not detected at Day 0, they were detected at later stages in the process, as noted below.

### Expression of adipogenic genes is dynamic throughout the differentiation process, and is influenced by 5-HT_2A_ receptor blockade

**Figure 6:**
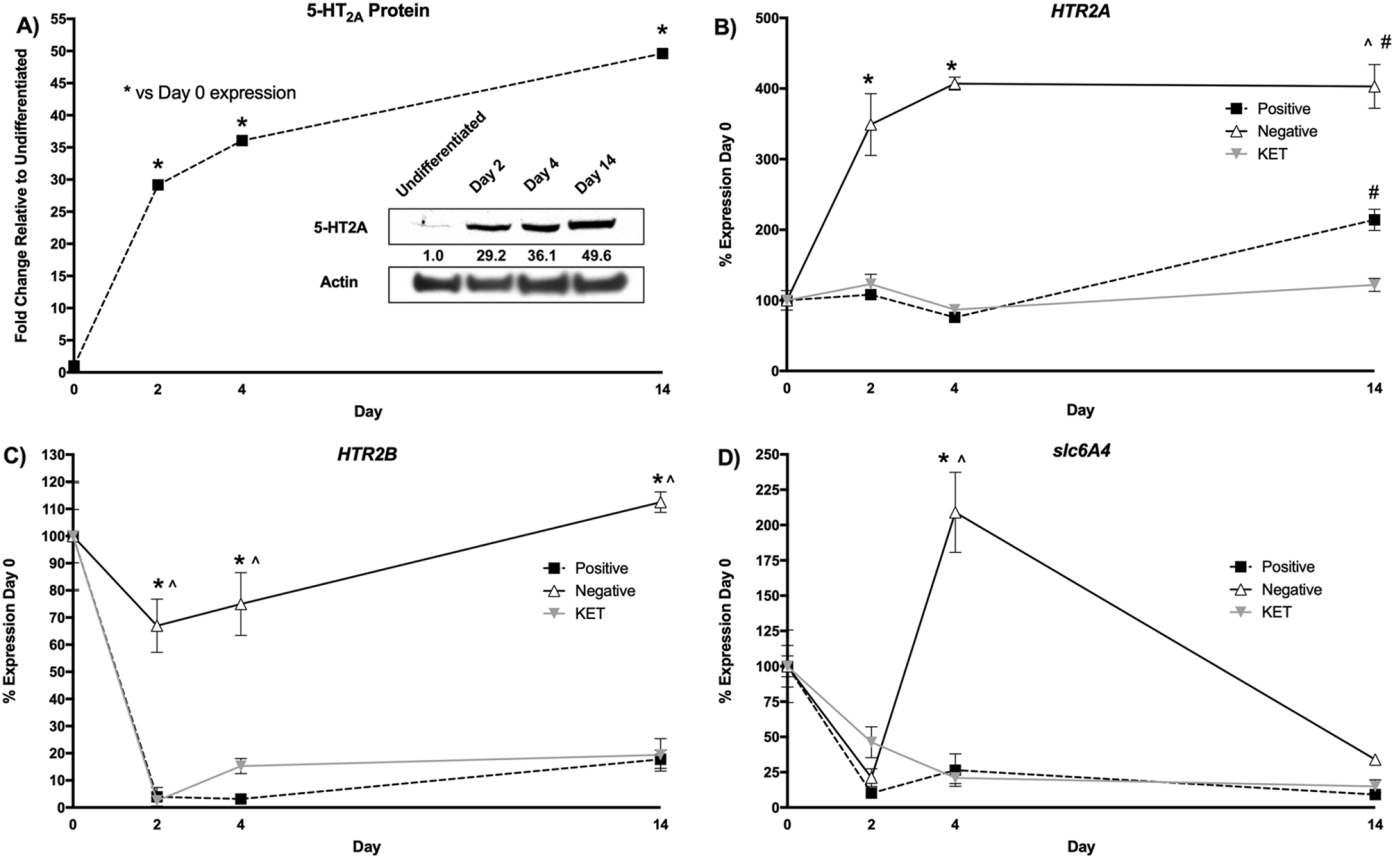
Dynamic protein and gene expression profile of transcripts for 5-HT_2A_, 5-HT_2B_ and SERT (serotonin transporter) during human adipocyte differentiation. (**A**) Levels of 5-HT_2A_ receptor protein determined by western blot during the differentiation process. The inset shows a representative western blot image, and the curve from the quantified data from the experiment. See Supplementary Figure S1 for the raw data corresponding to the western blot inset. (*p < 0.01 vs. negative control; n = 3 biological replicates per treatment; analysis of variance (ANOVA) with Tukey’s post hoc test). (**B**) Levels of 5-HT_2A_ receptor mRNA. (**C**) Levels of 5-HT_2B_ receptor mRNA, (**D**) levels of SERT mRNA. Positive represents expression from controls that were induced to differentiate. Negative represents controls that were not induced, and maintained in basal medium for 14 days. KET represents induced cells that were treated with 300 nM of Ketanserin at each step of the adipocyte differentiation process. (*p < 0.01 vs. negative control; #*p* < 0.01 vs. ketanserin treatment group; n = 3 biological replicates per treatment, with 3 technical replicates per biological replicate per gene expression data point; analysis of variance (ANOVA) with Tukey’s post hoc test). Ketanserin blocks the differentiation-induced expression of the 5-HT_2A_ receptor transcript, but has no effect on the 5-HT_2B_ receptor or SERT transcripts.

To elucidate molecular mechanisms mediating the influence of 5-HT_2A_ receptors, expression of different adipocyte markers was profiled during each major step of the differentiation process. As shown in Fig. 6, adipocyte related transcripts show dynamic patterns of expression throughout the differentiation process.

C/EBP-β and PPAR-γ are key transcription factors that control the adipocyte differentiation process^32,35^. These factors up-regulate downstream genes such as those for *adipocyte lipid binding protein* (*aP2*), and *adiponectin*, which lead to triglyceride elevation, lipid droplet accumulation, and a fully differentiated adipocyte phenotype. The aP2 protein is a commonly used molecular marker of adipocyte differentiation^28^. We found a dramatic increase in *aP2* expression in the positive induced control cells at day 2 that persisted through day 4, and ketanserin treatment blocked this expression increase (Fig. 5A).

For *c/ebp-β*, we observed an initial trend for increased expression both in the positive control as well as the ketanserin treated cells, compared with the negative control (Fig. 5B). Whereas *c/ebp-β* expression was maintained in the positive control cells throughout the process, both the negative control and the ketanserin treated cells showed parallel decreases to day 14.

For *ppar-γ*, there was an increase in expression in the positive control starting from day 2 that reached its peak at day 14 (Fig. 5C). There was very low expression of *ppar-γ* in the negative control. Significantly, expression increases of *ppar-γ* were blocked in induced cells by ketanserin treatment (Fig. 5C). Adiponectin is an adipokine secreted by differentiated adipocytes that clinical studies suggest correlates with levels of oxidative stress^38^. Further, high levels of adiponectin are observed in patients with metabolic syndrome or cardiovascular disease^38,39^. Significantly, transcription of *adiponectin* can be directly activated by *c/ebp-β*^40^. We found increased expression of *adiponectin* in the positive control cells beginning on day 2, increasing through day 14, and ketanserin treatment blocked *adiponectin* expression (Fig. 5D).

Sgk-1 activity is a key component of the adipocyte differentiation process. Inactivation of sgk-1 by siRNA-mediated knockdown of expression in the 3T3-L1 model blocks adipocyte differentiation^41^. Interestingly, we previously found that the powerful psychedelic drug, LSD, up-regulates *sgk-1* expression in rat brain cortex through activation of 5-HT_2A_ receptors^42^. In adipocyte differentiation, sgk-1 activity may be downstream of PPAR-γ activation^43^. Here, we have found a large increase in *sgk-1* expression in the positive control cells starting at day 2 that reached its peak at day 14 (Fig. 5E). The increases of *sgk-1* expression at day 14 also were blocked by ketanserin (Fig. 5E). Expression in the negative control was increased to a much greater extent than the positive control at day 2, and decreased somewhat through day 14 (Fig. 5E).

We examined levels of 5-HT_2A_ receptor protein over the differentiation process by western blot. We found that there was a low level of expression in undifferentiated preadipocytes, but that through the differentiation process, expression dramatically increased (Fig. 6A). These data were in agreement with the examination of 5-HT_2A_ receptor mRNA (*HTR2A*), where mRNA expression increased dramatically throughout the differentiation process (Fig. 6B). In the positive uninduced controls, *HTR2A* was not significantly changed until day 14, where it was increased only twofold. *HTR2A* in the ketanserin treated cells mirrored expression of the positive uninduced control until day 14, where the even twofold increase was blocked (Fig. 6B). In contrast to *HTR2A*, for 5-HT_2B_ (*HTR2B*) mRNA there was a small decrease in the negative control starting from day 2 to day 4, that returned to baseline levels at day 14 (Fig. 6C). In the positive differentiating control, there was a dramatic down-regulation of *HTR2B* expression (Fig. 6C). Ketanserin treatment had little effect on *HTR2B* expression when compared to the positive control (Fig. 6C). For the serotonin transporter *SERT*, there was dramatic down-regulation under all conditions at day 2, which remained lower at days 4 and 14 for both the positive control and ketanserin treated cells (Fig. 6D). Interestingly, the negative control had a significant upregulation of expression at day 4 that returned to positive control levels by day 14 (Fig. 6D).

## Discussion

Obesity is a leading worldwide public health concern that significantly affects not only individuals, but also their families^44^. It is now widely accepted that obesity contributes to a variety of diseases, including metabolic syndrome, diabetes, and atherosclerosis^45,46^. Increases in adipose tissue mass, as well as dysfunction of adipocyte differentiation, are tightly linked to obesity. Adipocyte differentiation is a highly regulated process, and understanding this process at a greater level of detail will lead to an enhanced understanding of adipocyte biology, and potentially to new therapeutic strategies to treat obesity and its related pathologies. Transcription factors such as C/EBP-β and PPAR-γ play very important roles in the regulation of adipocyte differentiation. During the differentiation of adipocytes, these transcription factors are activated, leading to increased expression of downstream factors that include aP2 and adiponectin^47^. Although a previous report^26^ has investigated 5-HT_2A_ receptor activity in the differentiation of mouse 3T3-L1 fibroblast cells to an adipocyte-like phenotype, they did not address potential mechanisms, and found that 5-HT_2A_ receptor activation *decreased* adipogenic biomarkers. Another report also purported to investigate the role of 5-HT_2A_ and 5-HT_2C_ receptors in 3T3-L1 differentiation and concluded that the 5-HT_2C_ receptor was involved^36^. Interestingly, they effectively demonstrated a role for the micro RNA, miR-488, in the differentiation process of 3T3-L1 cells through the use of siRNA, and proposed its regulation of 5-HT_2C_ receptor expression as a primary contributing mechanism to differentiation^36^. Because we can not detect the presence of 5-HT_2C_ receptor mRNA in *human* preadipocytes, or block the differentiation process with 5-HT_2C_ selective antagonists at relevant levels, the role of serotonin in the differentiation process of mouse 3T3-L1 cells from a fibroblast phenotype to an adipose-like phenotype is different from human preadipocyte to adipocyte differentiation such that 5-HT_2A_ receptor activity is required in humans but not in mice for proper adipocyte differentiation. A more recent study using RNA-Seq identified a potential role for 5-HT_2A_ receptors in the differentiation of bovine adipocytes, and implicated the PI3K-AKT pathway in this process^48^.

To establish that 5-HT_2A_ receptor activity is essential for differentiation of primary human preadipocytes, we first used two 5-HT_2_ receptor ligands at relevant concentrations to determine whether activation or blockade affected differentiation. Whereas the high affinity 5-HT_2_ receptor selective agonist (*R*)-DOI had no effect, the 5-HT_2A_ receptor antagonist ketanserin blocked adipocyte differentiation dose dependently. We validated these results using a second 5-HT_2A_ receptor selective antagonist, M100907. Although the 5-HT_2B_ receptor selective antagonist SB204741 blocked differentiation, the high concentration necessary to inhibit differentiation supports the notion that the effects of ketanserin, M100907, and SB204741 are all likely due to blockade of 5-HT_2A_ receptors, but we can not definitively rule out a contribution to the differentiation process by 5-HT_2B_ receptors. To validate our pharmacological studies, we used siRNA directed against 5-HT_2A_ receptor mRNA to knock down expression. Significantly, knockdown of 5-HT_2A_ receptor mRNA resulted in complete blockade of differentiation. Together, both pharmacological and genetic methods demonstrate that 5-HT_2A_ receptor activity is necessary for human adipocyte differentiation.

That serotonin receptor antagonists can block the differentiation process suggests that the process relies upon either serotonin present in the cell culture media, or in vivo, on circulating serotonin. The affinity of serotonin for the human 5-HT_2A_ receptor is higher than for rodent 5-HT_2A_ receptors (human: ~ 125 nM; rat ~ 1.1 µM; PDSP database). Typical cell culture media containing 10% FBS has a serotonin content of ~ 350 nM^49,50^. Therefore, there is likely enough serotonin present in the media to facilitate activation of the receptors and differentiation under normal culturing conditions. Interestingly, our gene expression results indicate the presence of serotonin transporter mRNA in adipose tissue, suggesting that adipocytes may themselves produce serotonin. Furthermore, adipose tissue has been shown to contain considerable amounts of serotonin^51^. It remains to be seen if putative ‘endogenous’ serotonin plays a significant role in adipocyte differentiation or function as it does in other peripheral cells like smooth muscle cells that synthesize their own serotonin and express SERT^14^.

How might 5-HT_2A_ receptor activity be required for the process of adipocyte differentiation? To answer this question we chose to examine the expression of genes previously implicated in adipocyte differentiation and function: *C/EBP-β*, *aP2*, *PPARγ*, *adiponectin*, and *sgk-1*^52,53^, and assess whether or not 5-HT_2A_ receptor activity influenced their expression. We examined gene expression at key time points throughout differentiation: Baseline (day 0), induction (day 2), differentiation (day 4), and maintenance/terminal differentiation (day 14). Expressions of all genes were dynamic throughout the process, and depending on when or if gene expression changes were affected by ketanserin, it might give clues to genes and pathways regulated by 5-HT_2A_ receptor activity during the differentiation process.

For expression of *CEBP-β* we found an initial trend for increased expression both in the positive control as well as the ketanserin treated cells, compared with the negative control. Whereas *CEBP-β* expression was maintained in the positive induced control throughout the differentiation process, for both the negative control and the ketanserin treated cells *CEBP-β* expression paralleled each other for a non-significant decrease at day 14. The absence of a ketanserin effect on *c/ebp-β* expression is consistent with 5-HT_2A_ activity likely acting downstream of *c/ebp-β* activation and we did find that ketanserin treatment dramatically influenced expression of genes known to be downstream of C/EBP-β activity.

Ketanserin treatment dramatically reduced *ppar-γ* expression, and subsequent gene expression of *aP2*, *adiponectin, and sgk-1*. Furthermore, the presence of the *ppar-γ* agonist rosiglitazone in the media in the induction phase abolished the inhibitory effect of ketanserin on adipocyte differentiation (data not shown). Taken together, we conclude that the role of 5-HT_2A_ activity on adipocyte differentiation occurs downstream of C/EBP-β but upstream of PPAR-γ.

Interestingly, we observed that mRNA levels of *HTR2A* were significantly increased in the differentiated adipocytes, and that mRNA levels for *HTR2B* dramatically decreased early in the process. One interpretation could be that the rapid decline in 5-HT_2B_ receptor expression and activity is facilitating differentiation. We do not believe that to be the case. Whereas ketanserin treatment blocks the expression increase in *HTR2A*, it has no effect on the decrease in *HTR2B*. If *HTR2B* expression changes were involved in mediating the effects of ketanserin, one would expect that ketanserin treatment would block the observed decrease.

We also observed that for some genes, including those for the serotonin receptors and transporter, as well as *sgk-1*, expression increased dramatically in negative control cells that were not induced. The differentiation process is initiated by the addition of certain chemicals to the media when the plates of cells just become confluent. It may be that as the plates become confluent, and contact between cells becomes prevalent, the cells induce certain mechanisms to inhibit growth (i.e. contact inhibition, growth arrest) that may be altering expression of these genes.

**Figure 7:**
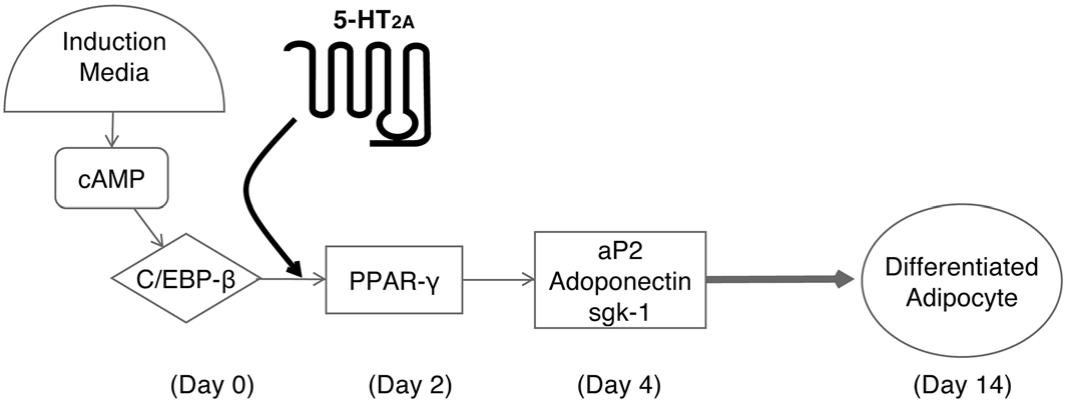
Model of adipocyte differentiation. Growth factors and second messengers lead to the production of C/EBP-β, which feeds into production of PPARγ and subsequent production of adipogenic factors including ap2, Adiponectin, and Sgk-1, whose action together leads to terminal differentiation of preadipose fibroblast cells to adipocytes. Activity of 5-HT_2A_ receptors is necessary for production and/or activity of PPARγ, downstream of C/EBP-β, to induce production of proadipogenic factors and terminal differentiation.

In summary, our data indicate that 5-HT_2A_ receptor activity is necessary for adipocyte differentiation in primary subcutaneous human preadipocytes. The 5-HT_2A_ receptor-mediated effects on adipocyte differentiation are likely mediated through modulation of expression of key genes necessary for the differentiation process. Based upon our data, we propose a model for the involvement of 5-HT_2A_ receptor activity in adipocyte differentiation as shown in Fig. 7. Growth factors and second messengers lead to the production of C/EBP-β, which feeds into production of PPARγ and subsequent production of adipogenic factors including ap2, Adiponectin, and Sgk-1, whose action together leads to terminal differentiation of preadipose fibroblast cells to adipocytes. Activity of 5-HT_2A_ receptors is necessary for production and/or activity of PPARγ, downstream of C/EBP-β, to induce production of proadipogenic factors and terminal differentiation. One possibility for the precise role of 5-HT_2A_ receptors is that the receptor activates a particular effector pathway that recruits or activates, or relieves inhibition of, components of transcriptional processes activated by *c/ebp-β* necessary for subsequent transcription of *ppar-γ*. Importantly, our results contribute to a greater understanding of the adipocyte differentiation process, and particularly the role of 5-HT_2A_ receptors in peripheral tissues, and this information may be relevant to the development of novel therapeutic strategies targeting this receptor for the treatment of obesity related diseases.

## Methods

### Reagents/chemicals

Standard cell culture media was provided by the Molecular and Cellular core of the Pharmacology Department at Louisiana State University Health Sciences Center; media supplements were purchased from Invitrogen (Carlsbad, CA, USA). The 5-HT_2A_ receptor antagonist ketanserin, 5-HT_2C_ receptor antagonist RS102221, and 5HT_2B_ receptor antagonist SB204741 were purchased from Tocris Bioscience (Ellisville, MO, USA). (*R*)-DOI and the selective 5-HT_2A_ receptor antagonist MDL100907 were gifts from Dr. David Nichols (Purdue University, West Lafayette, IN, USA). Isobutyl-methylxanthine (IBMX), dexamethasone, and insulin were purchased from Sigma-Aldrich (St. Louis, MO, USA).

### Cell culture

Primary human subcutaneous pre-adipocytes were purchased from Zen-bio, Inc (Research Triangle Park, NC, USA), and from two separate lots (#L120116E and #L050906). Individual experiments were performed with cells from only one lot, and not mixed. Each lot consists of preadipocytes from a single consenting Caucasian human female donor (non-smoker) with BMIs of 26.5 and 27.4, and ages of 52 and 46 years, respectively. As the source of human preadipocytes used in this study were from a narrow demographic, the possibility remains that preadipocytes isolated from different ages, sexes, BMIs, and races may have differential requirements for serotonin and 5-HT_2A_ receptor activity in their differentiation.

Primary human preadipocytes utilized for our studies were at passage 3. All cells were grown in tissue culture treated 6-well culture dishes (Corning, Lowell MA). The following cell culture media were used for the differentiation assay: Basal Medium (BM) = DMEM + 10% FBS; Induction Medium (IM) = DMEM + 10% FBS + 0.5 mM 3-isobutyl-1-methylxanthine (IBMX) + 10 μg/mL insulin + 1 μM dexamethasone; Maintenance Medium (MM) = DMEM + 10% FBS + 10 μg/mL insulin. Cells were grown to 100% confluence in BM. Two days after full confluency, cells were transferred to IM for 2 days, followed by MM for 2 days. The cells were then maintained in BM and grown for 10 days with medium change every 2 days. At the end of 10 days in BM, control differentiated cells demonstrated a change in morphology (increased lipid-loading compared to fibroblast-like morphology of preadipocytes) and Fat Oil red O staining of the lipid droplets.

### siRNA knockdown

Human subcutaneous pre-adipocytes were incubated in basal medium until ~ 50% confluent. Cells were transfected with siRNA against 5-HT_2A_ (siGENOME SMARTpool; Dharmacon, Lafayette, CO) and scrambled siRNA controls (siGENOME Non-targeting siRNA Pool #1; Dharmacon) using Fugene HD (Roche Diagnostics, Indianapolis, IN, USA) following manufacturers directions. After 48 h, cells were either induced to differentiate, or not induced depending on the treatment condition. At day 14, oil red O staining was performed to quantitate differentiation.

### Oil-red-O staining and microscopy

To determine the status of adipocyte differentiation, cells were washed twice with phosphate-buffered saline (PBS, pH 7.4), then fixed with 10% formalin at room temperature for 1 h, and stained with 0.5% oil-red-O solution for 1 h. After staining, the cultures were rinsed several times with 70% ethanol and images were taken using a Motic Images Plus CCD microscope camera and software (Richmond, BC, Ca). In order to determine the extent of adipocyte differentiation, the dye-triglyceride complex was extracted with 100% isopropanol and absorbance measured at 510 nM in a spectrophotometer^54–56^.

### RNA isolation and quantitative real-time polymerase chain reaction

RNA was extracted from cells using the Illustra RNAspin Mini kit from GE Healthcare Life Sciences (Piscataway, NJ, USA) following protocols supplied by the manufacturer. First-strand cDNA was generated using the ImProm-II cDNA synthesis kit (Promega, Madison, WI, USA) following the manufacturer's protocols using 300 ng total RNA. Quantitative real-time polymerase chain reaction (PCR) was performed using the ProbeLibrary system from Roche Diagnostics (Indianapolis, IN, USA) in combination with the HotStart-IT Probe qPCR Master Mix from USB (Cleveland, OH, USA) following the manufacturer's protocols. Primer sequences for each probe-based qPCR assay designed using Universal Probe Library Probe Finder software (Roche). Primer oligonucleotides were ordered from IDT (Coralville, IA). Corresponding probes were utilized from the Universal Probe Library (Roche). The sequences of primers and probes used for quantitative PCR are shown in Table 1. For the siRNA knockdown expression experiment, the IDT PrimeTime QPCR assay directed against the human HTR2A gene was used. For all quantitative determination of gene expression levels, reactions were performed using a two-step cycling protocol on a MyIQ-5 Cycler (Bio-Rad, Hercules CA, USA). Relative gene expression levels were calculated using the 2[− ΔΔC(T)] method^57^. Levels of all targets from the test samples were normalized to human *GUSB* expression.

**Table 1:** Gene and primer/probe information.

Gene	Accession	F/R	Sequence	UPL#
*aP2*	NM_001372066	Forward	5′-GGATGATAAACTGGTGGTGGA-3′	U85
		Reverse	5′-CACAGAATGTTGTAGAGTTCAATGC-3′	
*c/ebp-β*	NM_005194	Forward	5′-CGCTTACCTCGGCTACCA-3′	U74
		Reverse	5′-ACGAGGAGGACGTGGAGAG -3′	
*ppar-γ*	NM_138711	Forward	5′-GACAGGAAAGACAACAGACAAATC-3′	U70
		Reverse	5′-GGGGTGATGTGTTTGAACTTG-3′	
*adiponectin*	NM_004797	Forward	5′-AGAGATGGCACCCCTGGT-3′	U85
		Reverse	5′-CACCGATGTCTCCCTTAGGA-3′	
*GUSB*	NM_000181	Forward	5′-GTGACGTCTGTGCAGTCAGC-3′	U49
		Reverse	5′-CAACTTAAGGTGCCAGGTGTC-3′	
*HTR2A*	NM_000621	Forward	5′-GAGGCTCGTGCATGTAATCC-3′	U75
		Reverse	5′-TGTGCCACAAGGATCAGA AA-3′	
*HTR2B*	NM_000867	Forward	5′-GGAGAAGAAGCTGCAGTATGCTA-3′	U83
		Reverse	5′-GGCAGGACATAGAACAAGTGG-3′	
*HTR2C*	NM_001256760	Forward	5′-CCGAGTCCGTTTCTCGTCTA-3′	U69
		Reverse	5′-TCGCGGGTGTTAGCTGAT-3′	
*SLC6A4*	NM_001045	Forward	5′-TCTCTTGGTCCGGGCTTT-3′	U18
		Reverse	5′-ATAGAAACGAGCCCGTGGTT-3′	
*sgk1*	NM_005627	Forward	5′-TCCTAGACTACATTAATGGTGGAGAG-3′	U38
		Reverse	5′-ATAGAAACGAGCCCGTGGTT-3′	

### Western blot

Cell lysates collected in triplicate at the indicated time points were normalized for protein content by bicinchoninic acid (BCA) assay, separated on 4–12% continuous gradient Bolt Bis–Tris Plus polyacrylamide gels, and transferred to nitrocellulose membranes. Blots were serially probed with primary antibodies to human 5-HT_2A_ receptor (Santa Cruz, sc166775; 1:500, overnight at 4C) and beta-actin (Sigma, A2228; 1:5,000, 1 h at RT) followed by HRP conjugated secondary antibodies (1:50,000, 1 h at RT). Protein bands were visualized by chemiluminescence (WesternBright Sirius, Advansta) and captured on X-ray film. Densitometric analysis was performed using ImageJ. Individual bands, or an area of representative background, were encompassed within a defined area using the selection tool and mean gray values for each were determined. The background mean grey value was subtracted from all band measurements, and values between samples were normalized by ratioing the background adjusted 5-HT_2A_ receptor value relative to corresponding actin values. Normalized levels of 5-HT_2A_ receptor at each time point post-differentiation were expressed relative to undifferentiated controls. See Supplementary Figure S1 for scans of the representative western blot experiment whose data is shown in Fig. 6A.

## Supplementary Information

Supplementary Information.
