# Supplementary material for: Serotonin 5-HT2A receptor activity mediates adipocyte differentiation through control of adipogenic gene expression
Source: Sci Rep. 2021 Oct 5;11:19714. doi: 10.1038/s41598-021-98970-1 (PMC8492876; doi:10.1038/s41598-021-98970-1)
Supplement: Supplementary file 1 — Supplementary Information. [file 41598_2021_98970_MOESM1_ESM.docx]

**Serotonin 5-HT_2A_ receptor activity mediates adipocyte differentiation through control of adipogenic gene expression**

**Short Title:** 5-HT_2A_ receptor activity mediates adipocyte differentiation

Bangning Yu, Diana M. Battaglia, Timothy P. Foster, and Charles D. Nichols *

Department of Pharmacology and Experimental Therapeutics,

Louisiana State University Health Science Center, New Orleans, LA, USA

**
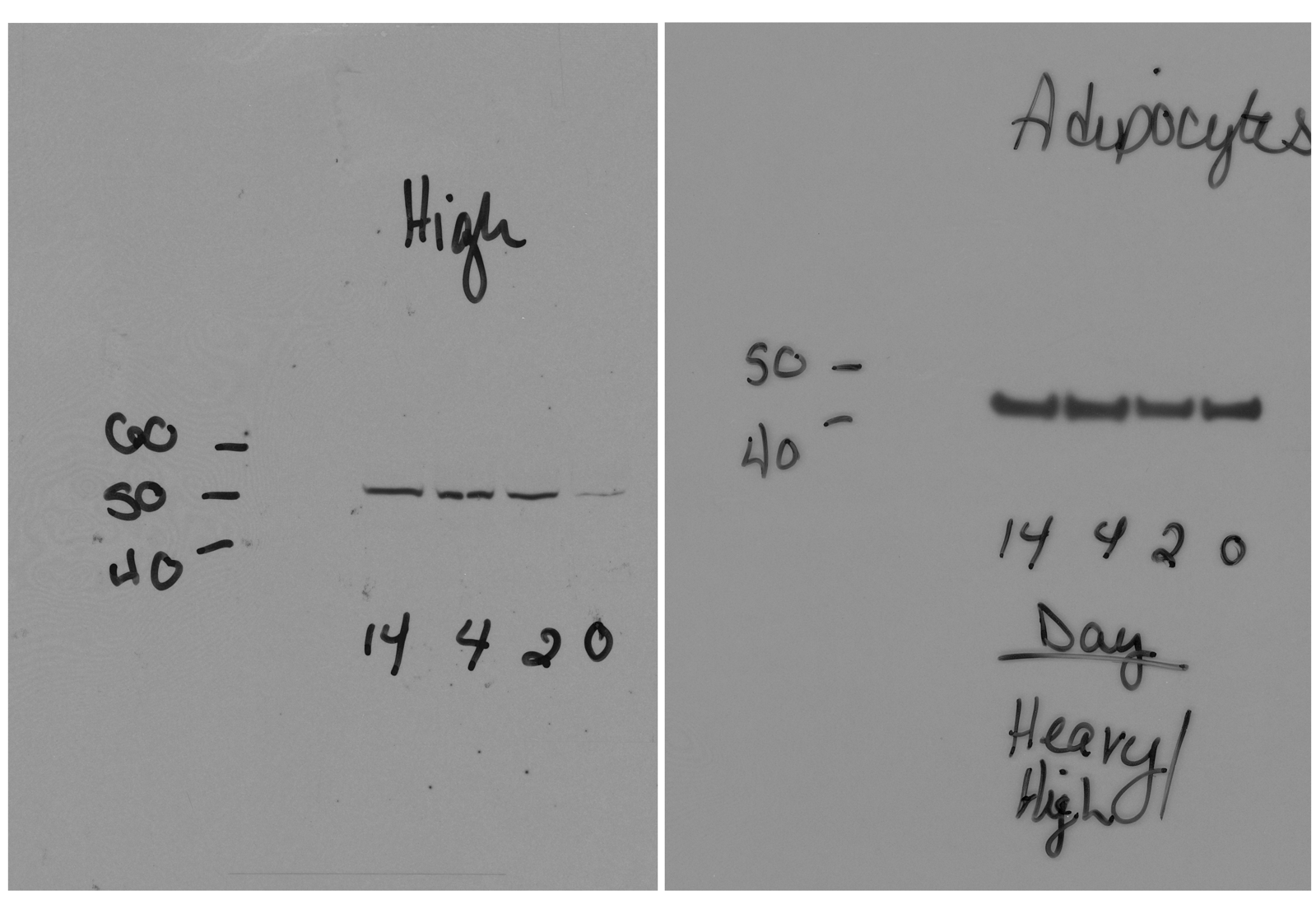
**

**Supplementary Figure 1.** Western blots of protein determination of 5-HT_2A_ receptor. **Left**) Scan of the membrane probed with antibody against 5-HT_2A_ receptor. Each lane is marked with the day of cell harvesting with relation to the differentiation process. **Right**) The membrane was stripped and re-probed with antibody against beta-actin. Each lane is marked with the day of cell harvesting with relation to the differentiation process.
